# S100A9 induces nucleus pulposus cell degeneration through activation of the NF‐κB signaling pathway

**DOI:** 10.1111/jcmm.16424

**Published:** 2021-03-18

**Authors:** Song Guo, Qihang Su, Junxiang Wen, Kai Zhu, Jun Tan, Qiang Fu, Guixin Sun

**Affiliations:** ^1^ Department of Orthopaedics Shanghai East Hospital Tongji University School of Medicine Shanghai China; ^2^ Department of Orthopaedics Shanghai General Hospital Shanghai Jiaotong University Shanghai China; ^3^ Department of Orthopaedics Ruijin Hospital Shanghai Jiaotong University Shanghai China; ^4^ Department of Traumatology Shanghai East Hospital Tongji University School of Medicine Shanghai China

**Keywords:** calcium‐binding S100A9 protein, cell apoptosis, inflammatory cytokines, Lumbar disc degeneration, matrix degradation, NF‐κB signalling pathway, oxidative stress

## Abstract

Oxidative stress in the lumbar disc leads to the degeneration of nucleus pulposus (NP). However, the molecular mechanisms underlying this process remain unclear. In this study, we delineated a key calcium‐binding protein, S100A9, which was induced by oxidative stress and was highly expressed in the degenerative NP. Immunofluorescence staining and Western blotting revealed that S100A9 induced NP cell apoptosis in vitro by up‐regulating the expression of pro‐apoptotic markers, including cleaved caspase‐3, cytochrome c and Bax. Moreover, RT‐PCR analyses revealed that the expression of S100A9 caused NP matrix degradation by up‐regulating the expression of matrix degradation enzymes and increased the inflammatory response by up‐regulating cytokine expression. Therefore, S100A9 induced NP cell degeneration by exerting pro‐apoptotic, pro‐degradation and pro‐inflammatory effects. The detailed mechanism underlying S100A9‐induced NP degeneration was explored by administering SC75741, a specific NF‐κB inhibitor in vitro. We concluded that S100A9 induced NP cell apoptosis, caused matrix degradation and amplified the inflammatory response through the activation of the NF‐κB signalling pathway. Inhibition of these pro‐apoptotic, pro‐degradation and pro‐inflammatory effects induced by S100A9 in NP may be a favourable therapeutic strategy to slow lumbar disc degeneration.

## 1 INTRODUCTION

Lower back pain (LBP) is a major public health problem worldwide and has become a huge socio‐economic burden.^1^, ^2^ It has been suggested that approximately 80% of individuals suffer from LBP in their life.^3^ Although the aetiology of LBP is complex, lumbar intervertebral disc degeneration (IDD) is regarded as the main cause of LBP.^4^, ^5^ Many factors, including genetic predisposition, smoking, infection, abnormal biomechanical loading and ageing, contribute to disc degeneration.^6^ The specific aims of the available therapy for lumbar disc degeneration are pain relief and symptom control. A systematic review by Phillips concluded that lumbar spinal fusion was an effective treatment strategy for patients who were refractory to non‐surgical treatments.^7^ However, many risks and complications are associated with lumbar spinal fusion surgery, including blood loss, dural sac tears and delayed recovery.^8^, ^9^ Therefore, further research on IDD pathogenesis to develop better therapy for IDD to slow disc degeneration is required.

The intervertebral disc (IVD) is composed of the central nucleus pulposus (NP), surrounding annulus fibrosus (AF), and the upper and lower cartilaginous endplates (CEPs).^10^ The NP is a highly hydrated tissue, which is majorly composed of aggrecan and type II collagen fibres, which function in absorbing the loads on the spine.^11^ NP structural deficits lead to the recruitment of immune cells to the NP, followed by the activation of the inflammatory cascade.^12^, ^13^ The increases in pro‐inflammatory cytokines, such as TNF‐α, IL‐1, IL‐6 and IL‐17, promote extracellular matrix degradation, chemokine production and changes in NP cell phenotype, which collectively accelerate NP degeneration.^14^ Additionally, the release of the above‐mentioned cytokines from degenerative NP further results in blood vessel ingrowth into the NP. Generally, the NP resides in a hypoxic microenvironment, which is essential for maintaining normal cellular metabolism and protein synthesis.^15^, ^16^, ^17^, ^18^, ^19^ Normally, blood vessels originate in the vertebral body and traverse the superficial region of the endplates; none of these vessels infiltrate the NP, except for some small discrete capillary beds in the dorsal and ventral surfaces.^17^ The presence of NP cells in a hypoxic microenvironment is reinforced by the low PO_2_ in the disc.^15^ Although NP cells have mitochondria with normal architecture, the total number of organelles per cell is low.^18^ NP cells are adapted to survive and grow in a hypoxic environment as indicated by the maximum disc cell survival at PO_2_ below 5% in vitro.^19^ Hypoxia plays a vital role in regulating the metabolism, function and fate of cells in the NP.^16^

Once the blood vessel grows into the NP and disrupts the hypoxic microenvironment, the subsequent oxidative stress induces NP degeneration. The expression of oxidative stress markers, including pentosidine and advanced glycation end products (AGEs), is increased in the degenerative human NP.^20^ Suzuki et al assessed the expression of nitrotyrosine, an oxidative stress marker, in a rat needle‐punctured disc degeneration model and human degenerative disc samples, and they found increased expression in the degenerative disc samples using immunohistochemistry and Western blotting.^21^ Furthermore, the researchers found that the administration of H_2_O_2_ into the cultured disc cells increased the expression of catabolic factors of disc degeneration, as assessed through RT‐PCR analysis, to reduce aggrecan levels. NAC, a known antioxidant, blocked MMP‐3 expression and down‐regulated aggrecan expression in both H_2_O_2_‐treated disc cells and a rat degenerative model. Excessive reactive oxygen species (ROS) was concluded to be a critical mediator in the pathogenesis of degenerative disc conditions and potential therapeutic target. Antioxidant NAC significantly abrogated the catabolic effect of excessive ROS in vitro and in vivo.^21^ Excessive nitric oxide (NO) in degenerative discs was related to extracellular matrix (ECM) degradation.^22^ The disruption of the hypoxic microenvironment in NP causes a failure in progenitor cell activation and a decrease in the number of NP cells, which leads to decreased cell function and enhancement of agents that promote disc degeneration.^23^ Additionally, Krupkova et al reported that EGCG administration protects human degenerative NP cells by inhibiting oxidative stress.^24^ However, the relationship between oxidative stress and the inflammatory cascade needs further elucidation.

Oxidative stress stimulates cells to produce a type of calcium‐binding protein named S100A9. When released from cells into the extracellular environment, S100A9 acts as an inflammatory cytokine exerting several effects on the target tissues. Studies have shown that increased levels of S100A9 at sites of inflammation influence the inflammatory cascade and the migration of myeloid cells.^25^, ^26^, ^27^, ^28^ S100A9 plays an essential role in the development of experimental osteoarthritis (OA), and S100A9 expression is differentially up‐regulated in chondrocytes, in the early stages of surgical induction in an OA model. Moreover, S100A9 caused a dose‐dependent down‐regulation of adult articular chondrocyte aggrecan and type II collagen levels by up‐regulating the expression of A distintegrin and metalloprotease with thrombospondin‐1 (Adamts1), A distintegrin and metalloprotease with thrombospondin‐4 (Adamts4), and A distintegrin and metalloprotease with thrombospondin‐5 (Adamts5), metalloproteinase‐1 (MMP‐1), matrix metalloproteinase‐3 (MMP‐3) and matrix metalloproteinase‐13 (MMP‐13) gene expression. This in vitro and in vivo study concluded that S100A9 accelerated cartilage matrix degradation and destruction in the early stages of OA by up‐regulating catabolic enzyme expression.^29^ Moreover, an increase in S100A9 in the synovium was observed in a collagenase‐induced osteoarthritis mouse model.^30^ Using a S100A9‐knockout mice, researchers demonstrated a major impact of S100A9 on OA cartilage destruction (45%‐73% inhibition) in comparison with wild‐type controls. Clinical trials also showed that OA patients had increased expression of S100A9 in the articular fluid, synovial membrane, blood and the damaged areas of the joint.^30^ Therefore, S100A9 is a key inflammatory cytokine that induces cartilage matrix breakdown and is involved in the pathogenesis of arthritis. Studies have also delineated that S100A9 induces apoptosis in multiple cancers.^31^, ^32^ Additionally, recombinant human S100A9 protein (rhS100A9) resulted in periodontium destruction by pro‐apoptotic and pro‐inflammatory effects on human periodontal ligament cells through the activation of the NF‐κB signalling pathway.^33^ Similarly, the activation of the NF‐κB signalling pathway induced an increase in the expression of matrix degradation genes, such as matrix MMP‐1, MMP‐3 and MMP‐13.^34^ Conversely, inhibition of the NF‐κB signalling pathway resulted in an increase in aggrecan by decreasing matrix degradation in a mouse disc degeneration model. This provides a promising therapeutic target for slowing disc degeneration.^34^ However, the detailed mechanism of NF‐κB signalling activation and the upstream functional molecules need further studies. S100A9 up‐regulated NF‐κB activity, as observed spectrophotometrically. It also increased cytokine expression and secretion, resulting in a strong pro‐inflammatory response in the human monocytic leukaemia cell line, THP‐1, and in the mouse bone marrow–derived dendritic cells.^35^ Treating human periodontal ligament cells (PDLCs) in vitro with recombinant human S100A9 (rhS100A9) and studying them using immunohistofluorescence indicated that rhS100A9 induces the nuclear translocation of NF‐κB p65 to increase pro‐inflammatory cytokine expression, including that of IL‐6, IL‐8, TNF‐α and COX_2_. Furthermore, blocking the NF‐κB pathway using PDTC, an NF‐κB inhibitor, successfully attenuated the rhS100A9‐induced cytokine up‐regulation, which confirms that S100A9 serves as an upstream regulator in the activation of the NF‐κB pathway in PDLCs.^33^ In case of periodontium destruction, S100A9 serves as an upstream inducer functioning through the activation of the NF‐κB signalling pathway. Therefore, we have been suggested that S100A9 also functions as an upstream cytokine that activates the NF‐κB signalling pathway, resulting in lumbar disc degeneration by inducing NP cell apoptosis and matrix degradation. Our preliminary data revealed increased expression of S100A9 in the degenerative NP tissue. In this study, we further investigated whether S100A9 induces NP cell apoptosis and matrix degradation by promoting matrix‐degrading enzyme expression and the inflammatory response. Additionally, the molecular mechanism of S100A9‐induced NP degeneration in vitro was studied.

## 2 MATERIALS AND METHODS

### 2.1 Ethical approval

This study was reviewed and approved by the Ethics Committee of Shanghai East Hospital, Tongji University School of Medicine. The study was performed in accordance with the Declaration of Helsinki in relation to research carried out on human participants. All patients and the next of kin were fully legally competent and consented to the use of lumbar NP tissues for research. Written informed consent from the patients or the next of kin was obtained. None of the patients belonged to a vulnerable population, and all patients or next of kin freely provided written informed consent. The privacy rights of the patients or next of kin were always protected. The patients or the next of kin provided written informed consent to publish case details in this manuscript.

## 3 PATIENTS AND TISSUE PREPARATION

All MRI images of the patients with a diagnosis of lumbar disc degeneration disease and lumbar vertebral fracture were assessed to determine the grade of lumbar disc degeneration by using the Pfirrmann degeneration grading system. HE staining was carried out to classify the grade of disc degeneration after collection of the NP tissues.

## 4 IMMUNOHISTOCHEMICAL DETECTION OF S100A9 EXPRESSION IN HUMAN NUCLEUS PULPOSUS TISSUES

The NP tissues were sectioned at a thickness of 5 µm. All sections were incubated for 1 hour with primary antibody directed against S100A9 (Cell Signaling) after blocking endogenous peroxidase by using 3% hydrogen peroxide for 5 minutes at 25℃. After rinsing, the sections were incubated for 1 hour with biotinylated horseradish peroxidase‐conjugated goat anti‐rabbit IgG (Abcam). Diaminobenzidine was used to develop peroxidase staining. Counterstaining was performed using haematoxylin (Abcam).

## 5 IMMUNOFLUORESCENCE STAINING TO DETECT CLEAVED CASPASE‐3 EXPRESSION IN NP TISSUES

The frozen sections were incubated in primary antibody against cleaved caspase‐3 (1/400, diluted in 1% BSA, 0.3% Triton X‐100; Cell Signaling) at 4°C overnight. After rinsing, the sections were placed in the dark and incubated in florescent‐labelled goat anti‐rabbit IgG (H&L Alexa Fluor 488, 1/500, diluted in 1% BSA, 0.3% Triton X‐100) for 1 hour. Then, Prolong Gold Antifade Reagent with DAPI was added to the sections.

## 6 EXTRACTION OF RNA AND REVERSE TRANSCRIPTION‐POLYMERASE CHAIN REACTION (RT‐PCR) ANALYSIS OF HUMAN NP TISSUE AND CULTURED CELLS

RNA from both NP tissue and cultured cells was extracted and was purified using a Qiagen Mini Kit. One microgram of total RNA was reverse‐transcribed, and complementary DNA was subjected to PCR. GAPDH was used as the normalizing gene. The mRNA expression levels of S100A9, matrix degradation enzyme genes (MMP‐3 and ADAMTS‐4), matrix genes (aggrecan and collagen‐II) and cytokines/chemokines (IL‐1, IL‐6, IL‐8 and TNF‐α) were measured and quantified using an ABI Prism 7000 Sequence Detection System. The primers used were as follows:

human MMP‐3 forward primer 5'‐TGAGGACACCAGCATGAACC‐3'.

and reverse 5'‐ACTTCGGGATGCCAGGAAAG‐3',

human ADAMTS‐4 forward primer 5'‐GAGGAGGAGATCGTGTTTCCA‐3'.

and reverse 5'‐CCAGCTCTAGTAGCAGCGTC‐3',

human Aggrecan forward primer 5'‐GTGCCTATCAGGACAAGGTCT‐3'.

and reverse 5'‐GATGCCTTTCACCACGACTTC‐3',

human collagen‐II forward primer 5'‐TGGACGATCAGGCGAAACC‐3'.

and reverse 5'‐GCTGCGGATGCTCTCAATCT‐3',

human IL‐1 forward primer 5'‐AGATGCCTGAGATACCCAAAACC‐3'.

and reverse 5'‐CCAAGCACACCCAGTAGTCT‐3',

human IL‐6 forward primer 5'‐CCTGAACCTTCCAAAGATGGC‐3'.

and reverse 5'‐TTCACCAGGCAAGTCTCCTCA‐3',

human IL‐8 forward primer 5'‐ACTGAGAGTGATTGAGAGTGGAC‐3'.

and reverse 5'‐AACCCTCTGCACCCAGTTTTC‐3',

human TNF‐α forward primer 5'‐GAGGCCAAGCCCTGGTATG‐3'.

and reverse 5'‐CGGGCCGATTGATCTCAGC‐3',

and human GAPDH forward primer 5'‐ACAACTTTGGTATCGTGGAAGG‐3'.

and reverse 5'‐GCCATCACGCCACAGTTTC‐3'.

## 7 HUMAN NP CELL ISOLATION AND CULTURE

Human NP tissue was harvested during discectomy surgery, isolated by collagenase digestion and grown to confluence in media containing serum for passage. Next, the cultured cells were validated by immunofluorescence staining with an aggrecan antibody (Cell Signaling), which is an NP cell‐specific marker.

## 8 APOPTOSIS ANALYSIS BY HOECHST 33 342 STAINING

After treating NP cells with 100 nM recombinant human S100A9 (rhS100A9) protein for 24 hours, the morphology of NP cells was examined by staining with Hoechst 33 342. At least 200 cells were randomly photographed and counted in both the rhS100A9 treatment and control groups. Cells with condensed and fragmented nuclei were regarded as apoptotic cells, and the percentages of apoptotic cells were calculated and compared between the different treatment groups.

## 9 IMMUNOFLUORESCENCE STAINING IN VITRO

After treatment with 100 nM rhS100A9 protein for 24 hours, NP cells were further processed for immunofluorescence staining detection of the pro‐apoptotic marker cleaved caspase‐3. Twenty thousand living cells were loaded onto each coverslip and incubated overnight. The coverslips were then incubated in 0.3% Triton X‐100, 1 × PBS and 5% NGD for 1 hour after 4% PFA fixation. After that, the fixed cells were incubated with primary antibodies, including cleaved caspase‐3 (1/400, diluted in 1% BSA, 0.3% Triton X‐100; Cell Signaling). After rinsing, the coverslips were placed in the dark and incubated in fluorescent‐labelled goat anti‐rabbit IgG (H&L Alexa Fluor 488, 1/500, diluted in 1% BSA, 0.3% Triton X‐100) for 1 hour. Then, the Prolong Gold Antifade Reagent with DAPI was added to the coverslips. All coverslips were randomly photographed, and at least 200 cells were counted. The percentage of positive cells was calculated and compared between the rhS100A9 treatment and control groups.

## 10 PROTEIN EXTRACTION AND WESTERN BLOTTING

After treatment with 100 nM rhS100A9 protein for 24 hours, NP cells were processed for protein extraction to detect the expression of pro‐apoptotic markers, including cleaved caspase‐3, cytochrome c and Bax, by using Western blotting. The attached NP cells were trypsinized and lysed using a lysis buffer (Bio‐Rad). Samples containing the denatured proteins from cell lysates were loaded into SDS‐PAGE gels and run for 1 hour at 100 V. The proteins were transferred to a polyvinylidene difluoride (PVDF) membrane (Bio‐Rad). After rinsing with 1 × TBST, the membranes were incubated in blocking solution (5% milk) for 1 hour at room temperature with constant rocking. After blocking, the membranes were incubated in primary antibody solution (1/1000 cleaved caspase‐3, 1/1000 cytochrome c and 1/1000 Bax) overnight at 4°C with gentle rocking. The membranes were washed with 1 × TBST three times for 10 minutes each with gentle rocking. The membranes were incubated with the appropriate diluted HRP‐conjugated secondary antibody (1/2000 in 1 × TBST) for 1 hour at room temperature with gentle rocking. The membranes were washed in 1 × TBST three times for 10 minutes each with gentle rocking. Immunodetection was performed using enhanced chemiluminescence (ECL) autoradiography film in a darkroom. Images were processed using the Geliance 200 Imaging System (Perkin‐Elmer, Waltham, MA, USA) and Gene Snap software (version 6.08.04; Syngene, Cambridge, UK) and were analysed by Gene Tools software (version 3.07.04; Syngene, Cambridge, UK).

## 11 NF‐κB SIGNALLING PATHWAY ACTIVATION EXAMINATION

To delineate the detailed mechanism by which S100A9 induces NP cell degeneration, we first tested the activation status of the NF‐κB signalling pathway. After treatment with 100 nM rhS100A9 protein for 6 hours, cultured NP cells were processed to detect the localization of P65 by using immunofluorescence staining. Additionally, we treated NP cells with 100 nM rhS100A9 protein and 5 µM SC75741, an NF‐κB‐specific inhibitor (MedChemExpress) for 6 and 12 hours to detect the protein expression of apoptosis markers, and the gene expression of inflammatory cytokines, matrix degradation enzymes and matrix by using Western blotting and qRT‐PCR. This was to investigate whether NP cell degeneration was reversed by blocking the NF‐κB signalling pathway.

### 11.1 Statistical analysis

Statistical analyses were performed by using SPSS (version 18.0; SPSS Inc, Chicago, IL, USA). The distribution of the variables was tested by using the Shapiro‐Wilk test. Count data are expressed as the mean ± standard deviation. Comparisons between multiple sets of means were performed by using one‐way analysis of variance (ANOVA). A value of *P* <.05 was considered statistically significant.

## 12 RESULTS

### 12.1 MRI Pfirrmann grade classification and HE staining of human NP tissue

To delineate the protein expression of S100A9 in human degenerative NP, we first determined the patients’ NP degenerative grades using MRI and then collected the NP tissue for HE staining. We analysed 40 patients with 60 lumbar disc segments by using the Pfirrmann grade classification. Grade III degeneration was the most common degeneration grade, as shown in the pie chart (Figure S1A). We further processed the NP tissues for HE staining and found a decreased number of NP cells and a disorganized extracellular matrix with increasing degrees of degeneration (Figure S1B).

## 13 INCREASED EXPRESSION OF S100A9 IN HUMAN DEGENERATIVE NP TISSUE RELATED TO NP CELL APOPTOSIS AND MATRIX DEGRADATION

**FIGURE 1:**
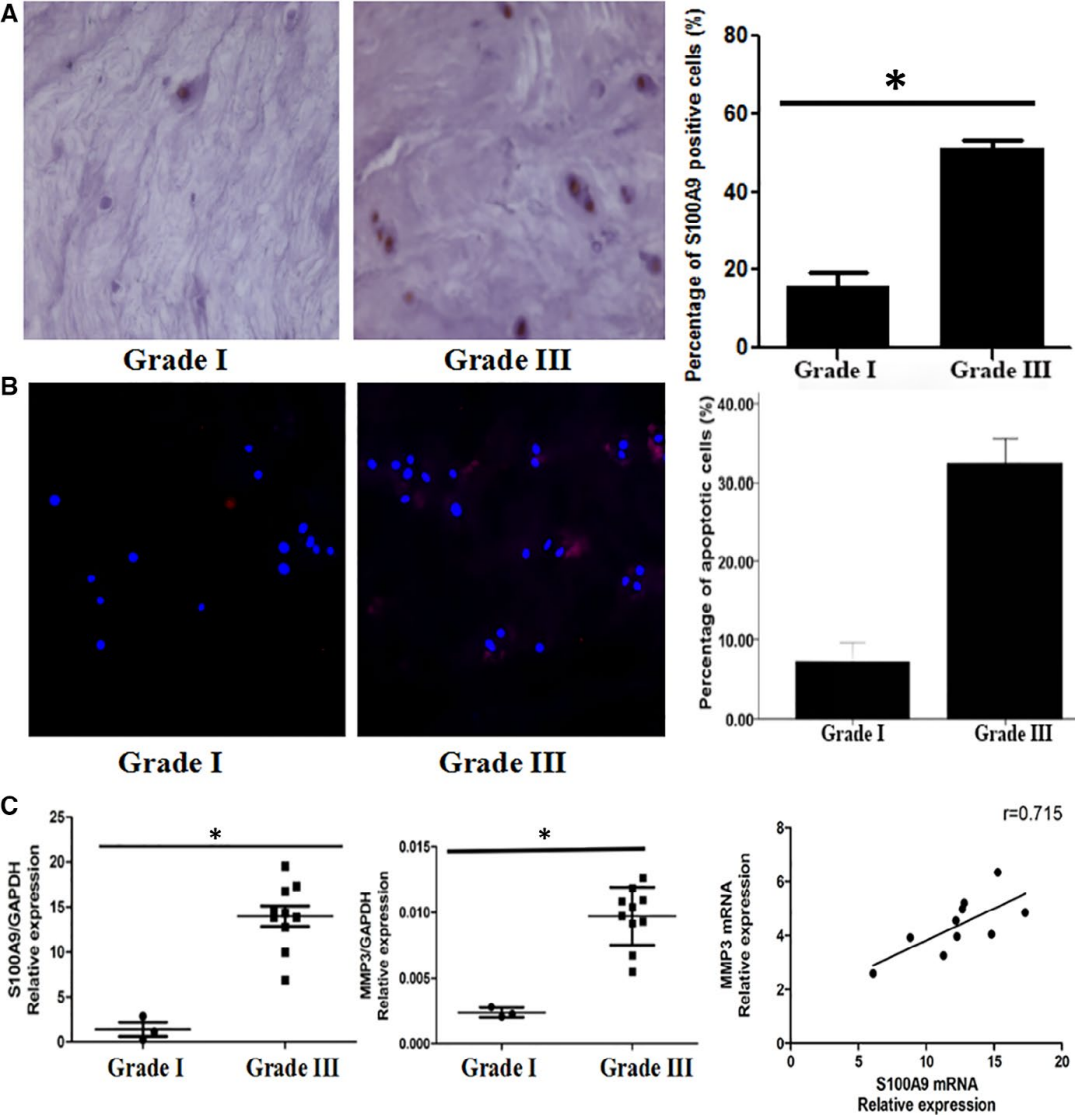
Increased expression of S100A9 in human degenerative nucleus pulposus (NP) tissue related to NP cell apoptosis and matrix degradation. Immunohistochemistry showed the percentage of S100A9‐positive cells in grade III NP was higher than that in grade I NP (A). Similarly, immunofluorescence showed the percentage of apoptotic cells (cleaved caspase‐3 positive cells) in grade III NP was 32.40% higher than that in grade I NP (B). The mRNA expression level of S100A9 in grade III nucleus pulposus was significantly higher than that in grade I. The mRNA expression of S100A9 in grade III was 10.14‐fold higher than that in grade I NP. Similarly, the mRNA expression level of MMP‐3 in grade III NP was significantly higher than that in grade I. The mRNA expression of MMP‐3 in grade III was 4.05‐fold higher than that in grade I NP. The mRNA expression levels of S100A9 and MMP‐3 in grade III NP specimens were positively correlated with the moderate correlation coefficient (r = 0.715) (C). MMP‐3 namely matrix metalloproteinase‐3, the most common matrix degradation enzyme that degrades lumbar disc matrix

After determination of the different degenerative grades, grade I and grade III NP tissues were examined for S100A9 protein expression using immunohistochemical staining. S100A9 protein is expressed in all NP cells. Comparative expression analysis showed increased expression of S100A9 in grade III NP tissue (Figure 1A). Additionally, a cellular apoptosis assay was performed to delineate the reason for the decreased number of NP cells by detecting the expression of cleaved caspase‐3, the most common apoptotic marker. Comparative expression analysis indicated an increase in apoptotic cells in grade III NP tissue when compared to that in grade I tissue (Figure 1B). qRT‐PCR was performed to investigate the gene expression of S100A9 and MMP‐3 in human NP tissue with varying degeneration grades. Gene expression of S100A9 and MMP‐3 was significantly increased in grade III NP tissue when compared to that of grade I tissue. The gene expression levels of S100A9 and MMP‐3 in grade III NP tissues were 10.41‐ and 4.05‐fold higher than those in grade I NP tissue, respectively. We also showed a positive correlation between the expressions of both genes in grade III degenerative NP tissue (Figure 1C).

## 14 PRO‐APOPTOTIC EFFECT OF RECOMBINANT HUMAN S100A9 (rhS100A9) PROTEIN ON NP CELLS

**FIGURE 2:**
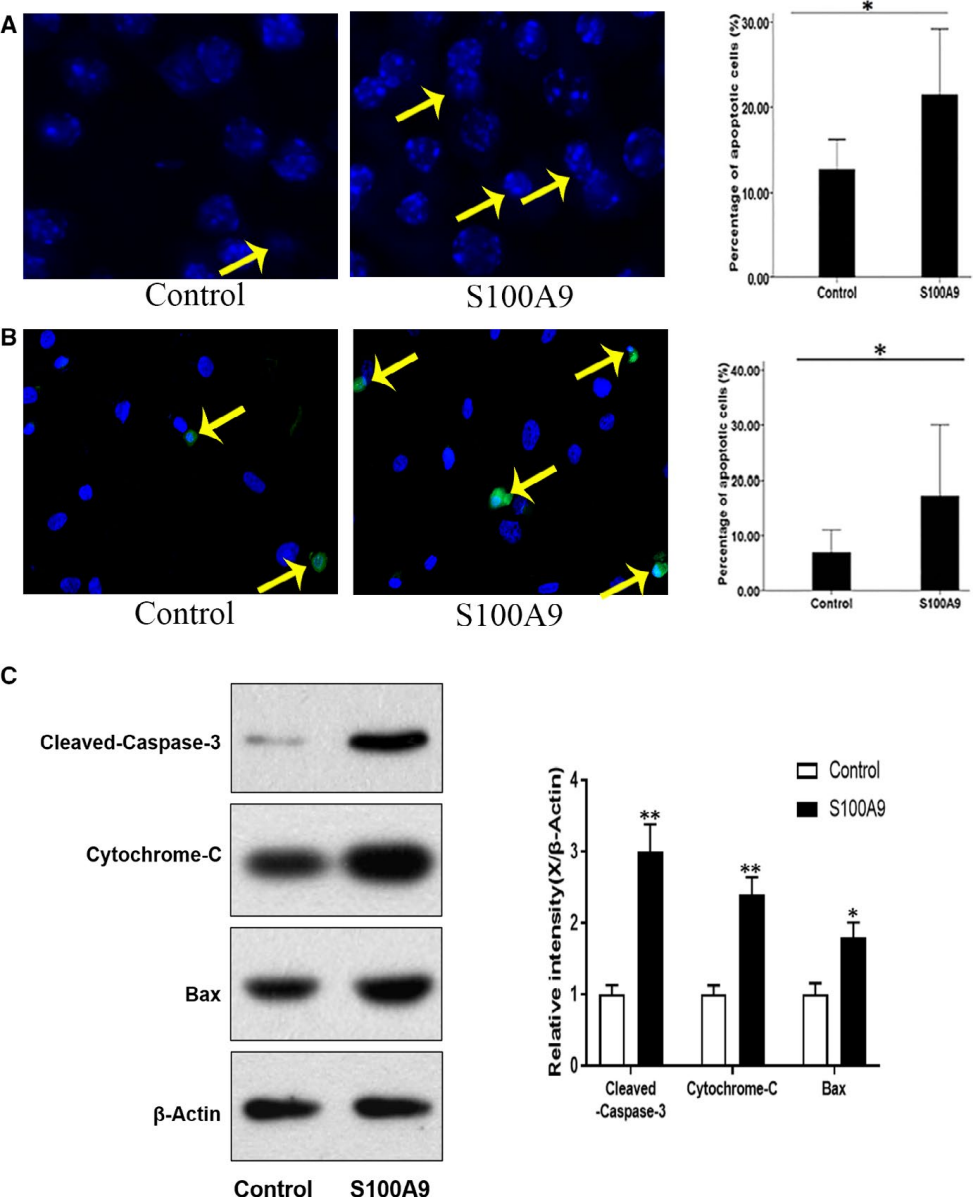
Pro‐apoptotic effect of recombinant human S100A9 (rhS100A9) protein on nucleus pulposus (NP) cells. Hoechst 33 342 fluorescence staining showed the increased proportion of nuclear dense granules in the human recombinant S100A9 protein (rhS100A9) treatment group than that in the control group (A). Additionally, immunofluorescence indicated the significant increase of cleaved caspase‐3–positive cells in the rhS100A9 treatment group than that in the control group (B). Western blotting also showed the increased expression of the pro‐apoptotic markers including cleaved caspase‐3, cytochrome c and Bax after rhS100A9 treatment of NP cells in vitro (C)

To determine the pro‐apoptotic effect of rhS100A9 on NP cells, a nuclear morphology assay and specific molecular marker detection assay were performed using Hoechst 33 342 and cleaved caspase‐3 staining, respectively. Apoptotic cells showed nuclear condensation and nuclear fragmentation after staining with Hoechst 33 342 dye. An increased proportion of nuclear condensation and fragmentation was observed in the rhS100A9 treatment group compared with that in the control group (Figure 2A). Additionally, the proportion of cleaved caspase‐3–positive cells was higher in the rhS100A9 treatment group (Figure 2B). Western blotting was performed to confirm the pro‐apoptotic effect of rhS100A9 on NP cells by detecting the expression of pro‐apoptotic markers, including cleaved caspase‐3, cytochrome c and Bax. After treatment with rhS100A9 protein, the levels of pro‐apoptotic proteins were significantly up‐regulated compared with those in the control group (Figure 2C).

## 15 PRO‐DEGRADATION AND PRO‐INFLAMMATORY EFFECTS OF RECOMBINANT HUMAN S100A9 PROTEIN ON NP CELL

**FIGURE 3:**
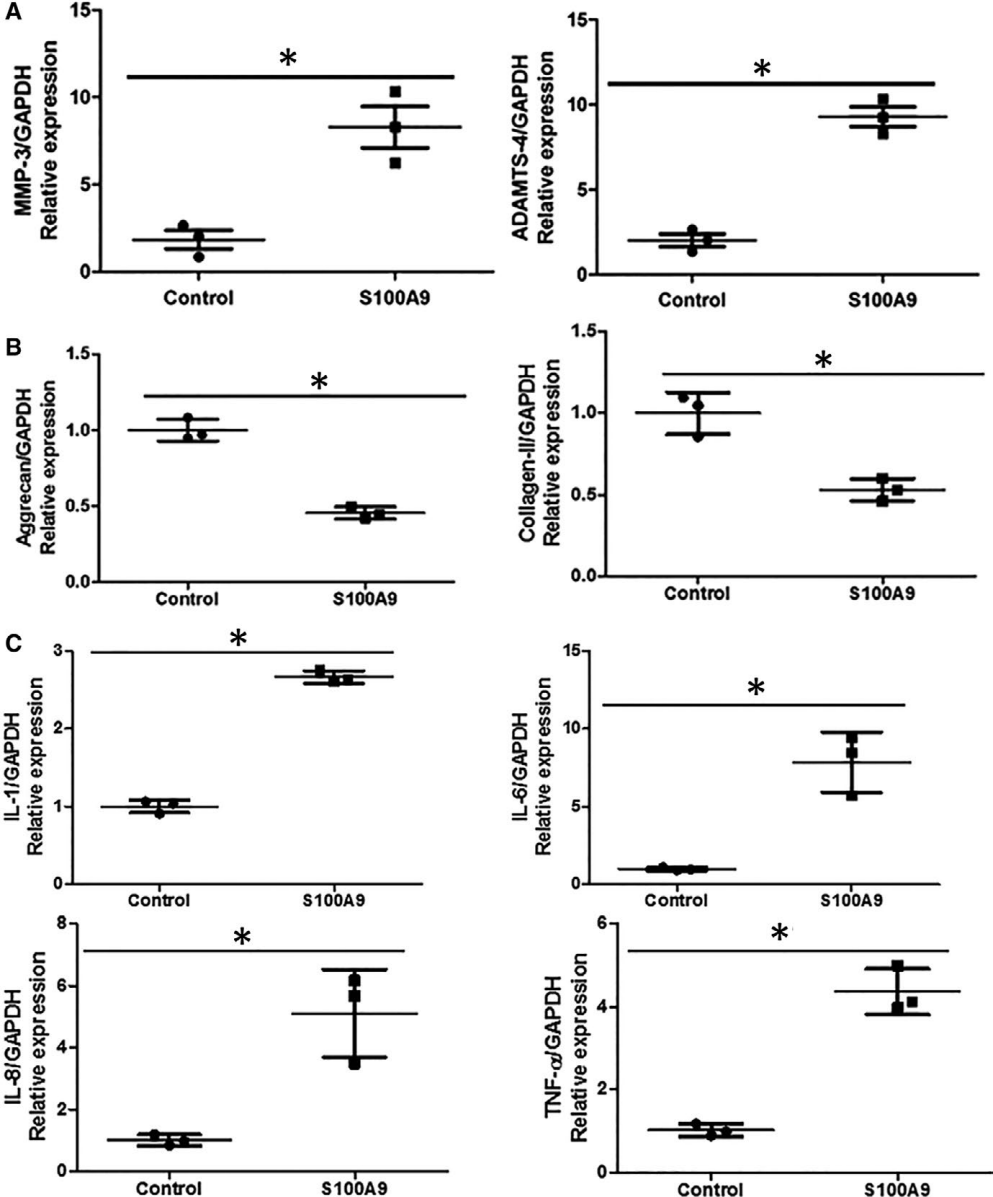
Pro‐degradation and pro‐inflammatory effects of recombinant human S100A9 protein on nucleus pulposus (NP) cells. The gene expression levels of MMP‐3 and ADAMTS‐4 in rhS100A9 treatment group were significantly increased by 4.49‐ and 4.62‐fold, respectively, compared with the control group (A), whereas the gene expressions of aggrecan and type II collagen in rhS100A9 treatment group were significantly decreased by approximately half when compared to the control group (B). rhS100A9 induced the up‐regulation of inflammatory cytokine genes including IL‐1, IL‐6, IL‐8 and TNF‐alpha. The gene expressions of IL‐1, IL‐6, IL‐8 and TNF‐α in rhS100A9 treatment group were significantly increased by 2.67‐, 7.85‐, 5.11‐ and 5.76‐fold respectively, compared with the control group (C). MMP‐3, matrix metalloproteinase‐3; ADAMTS‐4, A distintegrin and metalloprotease with thrombospondin‐4; IL‐1, interleukin‐1; IL‐6, interleukin‐6; IL‐8, interleukin‐8; TNF‐α, tumour necrosis factor‐alpha

To determine whether rhS100A9 causes matrix degradation, qRT‐PCR was performed to detect the gene expression of the common matrix degradation enzymes, namely MMP‐3 and ADAMTS‐4, and matrix‐protein genes, including aggrecan and type II collagen. After administration of rhS100A9 for 12 hours, the expression of matrix degradation enzyme in NP cells was significantly up‐regulated, whereas matrix‐protein gene expression was significantly down‐regulated compared with that in the control group (Figure 3A and B). The gene expression levels of MMP‐3 and ADAMTS‐4 in the rhS100A9‐treated group were 4.49‐ and 4.62‐fold higher than those in the control group, respectively, whereas the gene expression levels of aggrecan and type II collagen were both reduced by approximately half compared with those in the control group. As S100A9 functions as a pro‐inflammatory cytokine regulator, we also examined the gene expression of inflammatory cytokines after NP cells were treated with rhS100A9 protein. Figure 3C shows the increased gene expression of IL‐1, IL‐6, IL‐8 and TNF‐α in the rhS100A9 treatment group compared with that in the control group (Figure 3C). The gene expression levels of IL‐1, IL‐6, IL‐8 and TNF‐α in the rhS100A9‐treated group were 2.67‐, 7.85‐, 5.11‐ and 5.76‐fold higher than those in the control group, respectively.

## 16 rhS100A9 PROTEIN INDUCES NP CELL DEGENERATION THROUGH ACTIVATION OF THE NF‐κB SIGNALLING PATHWAY

**FIGURE 4:**
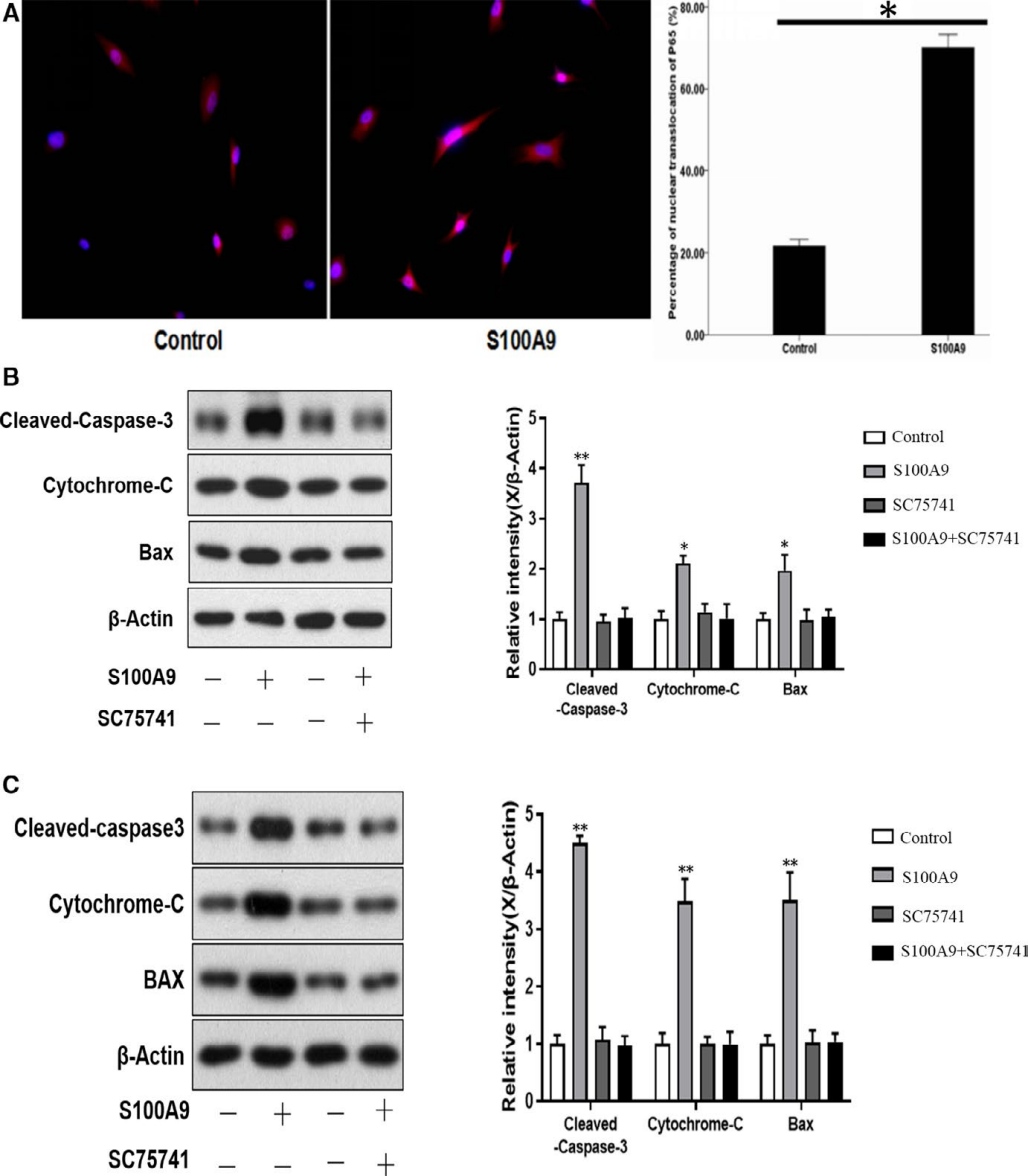
rhS100A9 activated NF‐κB signalling pathway by enhancing nuclear translocation of NF‐κB large subunit, P65. Immunofluorescence showed that P65 was expressed both in the cytoplasm and in the nuclei of NP cells. Comparative analysis indicated the increased expression of P65 in the cell nuclei of NP cells after treatment with rhS100A9 for 6 hours (A). Inhibition of NF‐κB signalling pathway demonstrated that rhS100A9 induced NP cell apoptosis through NF‐κB signalling pathway. The increased expressions of the pro‐apoptotic protein markers that were induced by rhS100A9 treatment for 6 hours and 12 hours were reversed by administration of SC75741, a NF‐κB specific inhibitor (B, C). NF‐κB, nuclear factor kappa‐B; NP, nucleus pulposus

**FIGURE 5:**
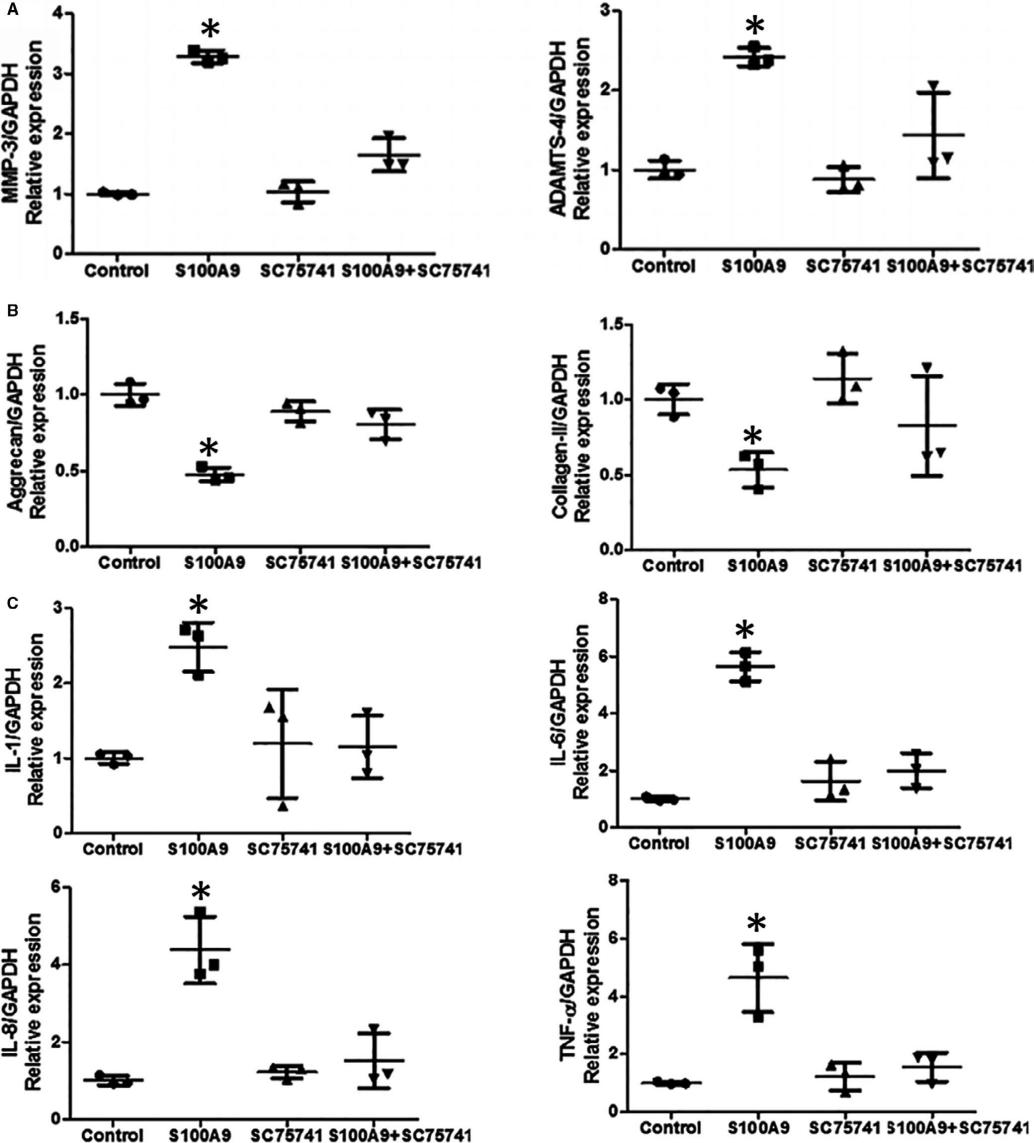
NF‐κB signalling pathway inhibition examination demonstrated rhS100A9 induced NP matrix degradation and inflammatory response through NF‐κB signalling pathway. The up‐regulated gene expressions of MMP‐3 and ADAMTS‐4 that were induced by the treatment of rhS100A9 for 12 hours were reversed by administration of SC75741 (A). The down‐regulated gene expressions of aggrecan and type II collagen that were induced by the treatment of rhS100A9 for 12 hours were reversed by administration of SC75741 (B). The up‐regulated gene expressions of IL‐1, IL‐6, IL‐8 and TNF‐α that were induced by the treatment of rhS100A9 for 12 hours were reversed by administration of SC75741 (C). NF‐κB, nuclear factor kappa‐B; NP, nucleus pulposus; MMP‐3, matrix metalloproteinase‐3; ADAMTS‐4, A distintegrin and metalloprotease with thrombospondin‐4; SC75741, a kind of NF‐kB–specific inhibitor; IL‐1, interleukin‐1; IL‐6, interleukin‐6; IL‐8, interleukin‐8; TNF‐α, tumour necrosis factor‐alpha

**FIGURE 6:**
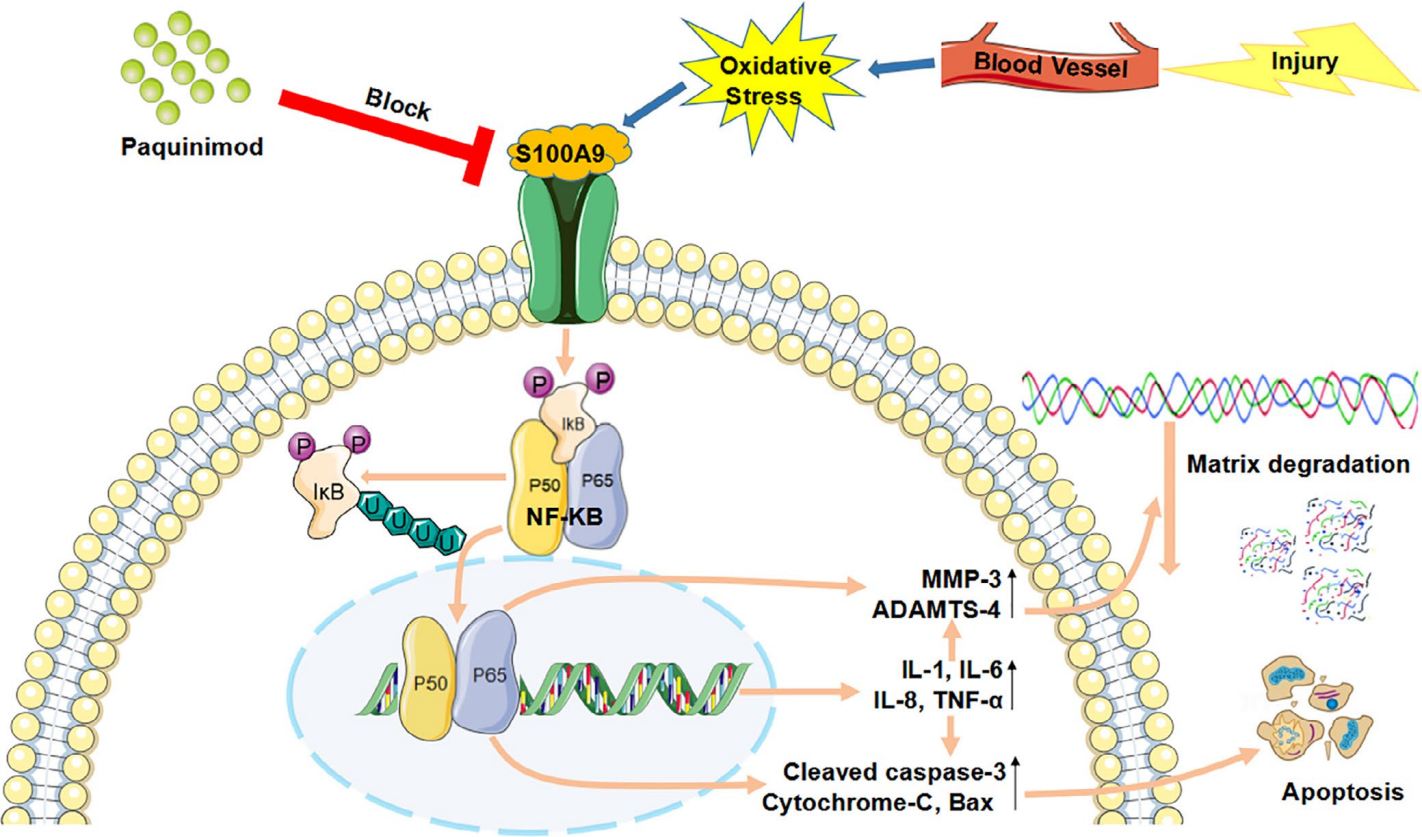
Schematic illustration of S100A9 inducing NP degeneration through the activation of NF‐κB signalling pathway. The injury of disc leads to the ingrowth of blood vessel into NP and the subsequent activation of the oxidative stress to induce the expression of S100A9 in NP cells. The expressed S100A9 combines with its specific receptor, TLR‐4, to induce P65 translocation into the cell nuclei to increase the expression of pro‐apoptotic, pro‐degradation and pro‐inflammatory genes, which results in the NP cell apoptosis and matrix degradation to accelerate NP degeneration. Paquinimod that was a specific S100A9 inhibitor can block the binding of S100A9 with TLR‐4. NP, nucleus pulposus; NF‐κB, nuclear factor kappa‐B; TLR‐4, Toll like receptor‐4

To investigate the mechanism by which S100A9 induces cell apoptosis, matrix degradation and the inflammatory response, immunofluorescence staining was performed to detect the nuclear translocation of p65, which functions as a subunit of NF‐κB. The presence of red fluorescence in the cell nucleus, which indicates NF‐κB translocation, typically indicates functional activation of p65 because it is otherwise retained in the cytoplasm in inactive complexes with IκB proteins. In untreated NP cells, p65 was almost exclusively localized in the cytoplasm. In contrast, rhS100A9 treatment induced the nuclear translocation of p65, as indicated by the pink nuclear signal in the merged images caused by overlapping of the blue (DAPI staining) and red fluorescence signals (NF‐κB‐p65 subunit) (Figure 4A). To confirm activation of the NF‐κB signalling pathway, we inhibited the NF‐κB signalling pathway by using the specific inhibitor, SC75741. This specific inhibitor attenuated rhS100A9‐induced NP cell apoptosis, as determined by quantitative assay of pro‐apoptotic markers (Figure 4B and C). Moreover, qRT‐PCR also showed that the pro‐degradation and pro‐inflammatory effects were both significantly reversed by administration of SC75741 (Figure 5). Collectively, it can be concluded that the pro‐apoptotic, pro‐degradation and pro‐inflammatory effects of rhS100A9 on NP cells occurred through activation of the NF‐κB signalling pathway (Figure 6).

## 17 DISCUSSION

In this study, we harvested human NP tissues and determined their degenerative grades by using MRI and HE staining. To determine S100A9 expression, we performed immunohistochemical staining with an S100A9 antibody and found increased expression of S100A9 in human degenerative NP. Moreover, we also demonstrated an increase in cell apoptosis in human degenerative NP by detecting the expression of cleaved caspase‐3, a common cell apoptotic marker.^36^ S100A9 functioned as an upstream regulator to induce periodontal ligament cell apoptosis, which led to the hypothesis that the increased expression of S100A9 was positively related to the expression of cleaved caspase‐3.^33^ Additionally, the gene expression of S100A9 and MMP‐3 in human NP was also explored using qRT‐PCR. We also found an increase in S100A9 and MMP‐3 gene expression and observed a positive correlation between the expressions of these two genes. A previous study showed that S100A9 induced dose‐dependent up‐regulation of MMP‐3 gene expression in adult articular chondrocytes and accelerated cartilage matrix degradation.^29^ Therefore, the increased expression of S100A9 in degenerative NP tissue may serve as a positive regulator of MMP‐3 and function in NP matrix degradation. Collectively, the increased expression of S100A9 in degenerative NP may be involved in NP cell apoptosis and matrix degradation to induce NP degeneration. To confirm the pro‐apoptotic and pro‐degradation effects of S100A9 on NP, we established an NP cell model in vitro by isolating human NP tissue.

NP cells were successfully cultured and validated by the detection of aggrecan expression, which is a NP cell–specific marker (Figure S2). When apoptosis occurred, cell nuclei become condensed and fragmented. The change in nuclear morphology was examined by using Hoechst 33 342 fluorescent dye.^37^ The detection of changes in nuclear morphology is also an effective method to determine cell apoptosis. Therefore, we calculated and compared the percentage of cells with condensed and fragmented nuclei after treatment of NP cells with recombinant human protein S100A9 (rhS100A9). It is well known that the increased expression levels of pro‐apoptotic markers, such as cleaved caspase‐3, cytochrome c and Bax, are also indicative of cell apoptosis.^36^, ^38^, ^39^ Therefore, we performed Western blotting and immunofluorescence staining to detect cleaved caspase‐3 expression after treating NP cells with rhS100A9. We confirmed that the administration of rhS100A9 in NP cell medium led to NP cell apoptosis by analysing the changes in the morphology and expression levels of cleaved caspase‐3. Additionally, the pro‐apoptotic effect of rhS100A9 was further confirmed by the increased expression of pro‐apoptotic markers, including cleaved caspase‐3, cytochrome c and Bax, as seen using Western blotting. Additionally, we determined that administration of rhS100A9 induced the up‐regulation of MMP‐3 and ADAMTS‐4, and the down‐regulation of aggrecan and type II collagen, using qRT‐PCR.

S100A9 functions as a pro‐inflammatory cytokine regulator that initiates the inflammatory response. We examined the pro‐inflammatory effect of S100A9 on NP cells.^40^, ^41^ We clearly demonstrated that rhS100A9 induced the up‐regulation of pro‐inflammatory cytokines such as IL‐1, IL‐6, IL‐8 and TNF‐α by detecting the expression of these genes. Increase in pro‐inflammatory cytokines accelerates NP degeneration by promoting extracellular matrix degradation, chemokine production and NP cell phenotype changes.^14^ In summary, we demonstrated that S100A9 induced NP cell degeneration by pro‐apoptotic, pro‐degradation and pro‐inflammatory effects. However, whether rhS100A9 directly induces NP cell apoptosis and matrix degradation or indirectly induces pro‐apoptotic and pro‐degradation effects by up‐regulating the inflammatory cytokines needs to be addressed in future studies.

To investigate the mechanism by which S100A9 induces NP cell degeneration, we first examined the activation of the NF‐κB signalling pathway because S100A9 is an upstream activator of the NF‐κB signalling pathway that results in periodontium destruction. Activation of the NF‐κB signalling pathway is characterized by the nuclear translocation of P65, a large NF‐κB subunit; thus, we performed immunofluorescence staining with a P65 primary antibody to determine the nuclear translocation of P65 after treating NP cells with rhS100A9.^42^, ^43^ The immunofluorescence results showed that P65 was located in both the cytoplasm and nucleus of NP cells. Comparative analysis showed an increase in nuclear P65 after treatment with rhS100A9, suggesting that rhS100A9 led to P65 translocation into the nucleus to activate downstream gene expression. Furthermore, we performed a rescue examination using SC75741, a specific NF‐κB inhibitor.^44^ The administration of this inhibitor successfully reversed the pro‐apoptotic, pro‐degradation and pro‐inflammatory effects induced by rhS100A9. Overall, we clearly delineated that S100A9 induced NP cell apoptosis and matrix degradation, and amplified the inflammatory response through the activation of the NF‐κB signalling pathway (Figure 6). In summary, S100A9 is an inflammatory cytokine that is highly expressed in human degenerative NP. Administration of rhS100A9 in vitro induced NP cell degeneration through activation of the NF‐κB signalling pathway. Inhibition of the pro‐apoptotic, pro‐degradation and pro‐inflammatory effects of S100A9 on NP cells may be a therapeutic strategy to slow disc degeneration.

This study has certain limitations that should be indicated and listed here. First, the NP cell apoptosis assay was not re‐confirmed using flow cytometry, which was rectified in our subsequent study. Second, although we indicated the pro‐apoptotic, pro‐degradation and pro‐inflammatory effects of rhS100A9 on NP cells, the detailed relationships among these effects were not explored in this study. Finally, as this was an in vitro study, all findings need to be validated by using a suitable animal model in the next study. We will use paquinimod, a specific S100A9 inhibitor, to investigate its potential effects in delaying NP degeneration in a well‐established rat disc degeneration model (Figure S3and Figure S4).

## 18 CONCLUSIONS

Lumbar intervertebral disc degeneration (IDD) causes severe chronic back and leg pain, which brings a great burden to patients and society. Lumbar spinal fusion surgery is an effective treatment strategy for patients who are refractory to non‐surgical treatments. However, many risks and complications relate to lumbar spinal fusion surgery, including blood loss, dural sac tears and accelerated degeneration of the adjacent segments. It has become a new research direction to slow down the progression of IDD from the molecular level by studying the pathogenesis of IDD. Recently, many studies focus on oxidative stress causing IDD, which has become a research hot spot of IDD. However, the specific molecular mechanism of oxidative stress–induced IDD has not been elucidated. We firstly discovered a calcium‐binding protein, namely S100A9 that was associated with oxidative stress in IDD could induce nucleus pulposus cells apoptosis, matrix degradation by up‐regulating the expression of matrix degradation enzymes and increase the inflammatory response by up‐regulating cytokine expression in vitro. Additionally, we clearly demonstrated that S100A9 exerted pro‐apoptotic, pro‐degradation and pro‐inflammatory effects on nucleus pulposus cells through the activation of the NF‐κB signalling pathway. This study partly clarified the molecular mechanism of oxidative stress–induced IDD.

## CONFLICT OF INTEREST

The authors declare that there are no conflict of interests.

## AUTHOR CONTRIBUTION

Song Guo: Conceptualization (lead); Data curation (lead); Resources (lead); Software (lead); Supervision (lead); Validation (lead). Qihang Su: Conceptualization (equal); Data curation (equal); Resources (equal); Software (equal); Supervision (equal). Junxiang Wen: Conceptualization (equal); Data curation (equal); Investigation (equal); Methodology (equal); Writing‐original draft (equal). Kai Zhu: Conceptualization (equal); Data curation (equal); Methodology (equal); Project administration (equal); Writing‐original draft (equal); Writing‐review & editing (equal). Jun Tan: Investigation (equal); Visualization (equal); Writing‐review & editing (equal). Qiang Fu: Formal analysis (equal); Funding acquisition (equal); Visualization (equal); Writing‐original draft (equal). Guixin Sun: Data curation (equal); Formal analysis (equal); Funding acquisition (equal); Resources (equal); Software (equal).

## Supporting information

Fig S1

Fig S2

Fig S3

Fig S4

## Data Availability

All data generated or analysed during this study are included in this published article and available from the corresponding author upon reasonable request.

## References

[1] Urits I , Burshtein A , Sharma M , et al. Low back pain, a comprehensive review: pathophysiology, diagnosis, and treatment. Curr Pain Headache Rep. 2019;23:doi:10.1007/s11916‐019‐0757‐1 10.1007/s11916-019-0757-1 30854609

[2] Karran EL , Grant AR , Moseley GL . Low back pain and the social determinants of health: a systematic review and narrative synthesis. Pain. 2020;161:2476‐2493. 32910100 10.1097/j.pain.0000000000001944

[3] Damian H , Christopher B , Gail W . A systematic review of the global prevalence of low back pain. Arthritis Rheum. 2012;64:2028‐2037. 22231424 10.1002/art.34347

[4] DePalma MJ , Ketchum JM , Saullo T . What is the source of chronic low back pain and does age play a role? Pain medicine. 2011;12:224‐233. 21266006 10.1111/j.1526-4637.2010.01045.x

[5] Chou D , Samartzis D , Bellabarba C , et al. Degenerative magnetic resonance imaging changes in patients with chronic low back pain: a systematic review. Spine. 2011;36:S43‐53. 21952189 10.1097/BRS.0b013e31822ef700

[6] Battié MC , Videman T , Parent E . Lumbar disc degeneration: epidemiology and genetic influences. Spine. 2004;29:2679‐2690. 15564917 10.1097/01.brs.0000146457.83240.eb

[7] Phillips FM , Slosar PJ , Youssef JA , et al. Lumbar spine fusion for chronic low back pain due to degenerative disc disease: a systematic review. Spine. 2013;38:E409‐E422. 23334400 10.1097/BRS.0b013e3182877f11

[8] Noriega DC , Ardura F , Hernández‐Ramajo R , et al. Intervertebral disc repair by allogeneic mesenchymal bone marrow cells: a randomized controlled trial. Transplantation. 2016;101:1945‐1951. 10.1097/TP.0000000000001484 27661661

[9] Pereira CL , Teixeira GQ , Ribeiro‐Machado C , et al. Mesenchymal stem/stromal cells seeded on cartilaginous endplates promote intervertebral disc regeneration through extracellular matrix remodeling. Sci Rep. 2016;6:1‐17. 10.1038/srep33836 27652931 PMC5031983

[10] Pattappa Z , Li M , Peroglio N , et al. Diversity of intervertebral disc cells: phenotype and function. J Anat. 2012;221:480‐496. 22686699 10.1111/j.1469-7580.2012.01521.x PMC3512276

[11] Roberts S , Menage J , Duance V , et al. Collagen types around the cells of the intervertebral disc and cartilage end plate: an immunolocalization study. Spine. 1991;16:1030‐1038. 1948394

[12] Shamji MF , Setton LA , Wingrove J , et al. Proinflammatory cytokine expression profile in degenerated and herniated human intervertebral disc tissues. Arthritis Rheum. 2010;62:1974‐1982. 20222111 10.1002/art.27444 PMC2917579

[13] Kokubo Y , Uchida K , Kobayashi S , et al. Herniated and spondylotic intervertebral discs of the human cervical spine: histological and immunohistological findings in 500 en bloc surgical samples. Laboratory investigation. J Neurosurg Spine. 2008;9:285‐295. 18928227 10.3171/SPI/2008/9/9/285

[14] Yamamoto J , Maeno K , Takada T , et al. Fas ligand plays an important role for the production of pro‐inflammatory cytokines in intervertebral disc nucleus pulposus cells. J Orthop Res. 2013;31:608‐615. 23192951 10.1002/jor.22274

[15] Bartels EM , Fairbank JCT , Winlove CP , et al. Oxygen and lactate concentrations measured in vivo in the intervertebral discs of patients with scoliosis and back pain. Spine. 1998;23:1‐7. 9460145 10.1097/00007632-199801010-00001

[16] Risbud MV , Schipani E , Shapiro IM . Hypoxic regulation of nucleus pulposus cell survival: from niche to notch. Am J Pathol. 2010;176:1577‐1583. 20133815 10.2353/ajpath.2010.090734 PMC2843446

[17] Gruber HE , Ashraf N , Kilburn J , et al. Vertebral endplate architecture and vascularization: application of micro‐computerized tomography, a vascular tracer, and immunocytochemistry in analyses of disc degeneration in the aging sand rat. Spine. 2005;30:2593‐2600. 16319744 10.1097/01.brs.0000187877.30149.83

[18] Gan JC , Ducheyne P , Vresilovic EJ , et al. Intervertebral disc tissue engineering I: characterization of the nucleus pulposus. Clin Orthop Relat Res. 2003;411:305‐314. 10.1097/01.blo.0000063796.98363.9a 12782889

[19] Risbud MV , Guttapalli A , Albert TJ , et al. Hypoxia activates MAPK activity in rat nucleus pulposus cells: regulation of integrin expression and cell survival. Spine. 2005;30:2503‐2509. 16284587 10.1097/01.brs.0000186326.82747.13

[20] Sivan S , Tsitron E , Wachtel E , et al. Age‐related accumulation of pentosidine in aggrecan and collagen from normal and degenerate human intervertebral discs. Biochem J. 2006;399:29‐35. 16787390 10.1042/BJ20060579 PMC1570172

[21] Suzuki S , Fujita N , Hosogane N , et al. Excessive reactive oxygen species are therapeutic targets for intervertebral disc degeneration. Arthritis Res Ther. 2015;17: 10.1186/s13075-015-0834-8 PMC4635526 26542776

[22] Hou G , Lu H , Chen M , et al. Oxidative stress participates in age‐related changes in rat lumbar intervertebral discs. Arch Gerontol Geriatr. 2014;59:665‐669. 25081833 10.1016/j.archger.2014.07.002

[23] Risbud MV , Schipani E , Shapiro IM . Hypoxic regulation of nucleus pulposus cell survival. Am J Pathol. 2010;176:1577‐1583. 20133815 10.2353/ajpath.2010.090734 PMC2843446

[24] Krupkova O , Handa J , Hlavna M , et al. The natural polyphenol epigallocatechin gallate protects intervertebral disc cells from oxidative stress. Oxid Med Cell Longev. 2016;2016:1‐17. 10.1155/2016/7031397 PMC4826942 27119009

[25] Shabani F , Farasat A , Mahdavi M , et al. Calprotectin (S100A8/S100A9): a key protein between inflammation and cancer. Inflamm Res. 2018;67:801‐812. 30083975 10.1007/s00011-018-1173-4

[26] Narumi K , Miyakawa R , Ueda R , et al. Proinflammatory proteins S100A8/S100A9 activate NK cells via interaction with RAGE. J Immunol. 2015;194:5539‐5548. 25911757 10.4049/jimmunol.1402301

[27] Foell D , Wittkowski H , Vogl T , et al. S100 proteins expressed in phagocytes: a novel group of damage‐associated molecular pattern molecules. J Leukoc Biol. 2007;81:28‐37. 16943388 10.1189/jlb.0306170

[28] Ehrchen JM , Sunderkotter C , Foell D , et al. The endogenous Toll‐like receptor 4 agonist S100A8/S100A9 (calprotectin) as innate amplifier of infection, autoimmunity, and cancer. J Leukoc Biol. 2009;86:557‐566. 19451397 10.1189/jlb.1008647

[29] Zreiqat H , Belluoccio D , Smith MM , et al. S100A8 and S100A9 in experimental osteoarthritis. Arthritis Res Ther. 2010;12:R16. 10.1186/ar2917 20105291 PMC2875644

[30] van Lent PLEM , Blom AB , Schelbergen RFP , et al. Active involvement of alarmins S100A8 and S100A9 in the regulation of synovial activation and joint destruction during mouse and human osteoarthritis. Arthritis Rheum. 2012;64:1466‐1476. 22143922 10.1002/art.34315

[31] Qin F , Song Y , Li Z , et al. S100A8/A9 induces apoptosis and inhibits metastasis of CasKi human cervical cancer cells. Pathol Oncol Res. 2010;16:353‐360. 19957061 10.1007/s12253-009-9225-2

[32] Ghavami S , Kerkhoff C , Los M , et al. Mechanism of apoptosis induced by S100A8/A9 in colon cancer cell lines: the role of ROS and the effect of metal ions. J Leukoc Biol. 2004;76:169‐175. 15075348 10.1189/jlb.0903435

[33] Zheng Y , Hou J , Peng L , et al. The pro‐apoptotic and pro‐inflammatory effects of calprotectin on human periodontal ligament cells. PLoS One. 2014;9(10):e110421. 10.1371/journal.pone.0110421 25338166 PMC4206420

[34] Wuertz K , Quero L , Sekiguchi M , et al. The red wine polyphenol resveratrol shows promising potential for the treatment of nucleus pulposus mediated pain in vitro and in vivo. Spine. 2011;21:E1373‐E1384. 10.1097/BRS.0b013e318221e655 21587103

[35] Riva M , Kallberg E , Bjork P , et al. Induction of nuclear factor‐kappaB responses by the S100A9 protein is Toll‐like receptor‐4‐ dependent. Immunology. 2012;137:172‐182. 22804476 10.1111/j.1365-2567.2012.03619.x PMC3461398

[36] Kothakota S , Azuma T , Reinhard C , et al. Caspase‐3‐generated fragment of gelsolin: effector of morphological change in apoptosis. Science. 1997;278:294‐298. 9323209 10.1126/science.278.5336.294

[37] Portugal J , Waring MJ . Assignment of DNA binding sites for 4',6‐diamidine‐2‐phenylindole and bisbenzimide (Hoechst 33258). A comparative footprinting study. Biochim Biophys Acta. 1988;949:158‐168. 2449244 10.1016/0167-4781(88)90079-6

[38] Wei MC , Zong WX , Cheng EH , et al. Proapoptotic BAX and BAK: a requisite gateway to mitochondrial dysfunction and death. Science. 2001;292:727‐730. 11326099 10.1126/science.1059108 PMC3049805

[39] Li P , Nijhawan D , Budihardjo I , et al. Cytochrome c and dATP‐dependent formation of Apaf‐1/caspase‐9 complex initiates an apoptotic protease cascade. Cell. 1997;91:479‐489. 9390557 10.1016/s0092-8674(00)80434-1

[40] Chernov AV , Dolkas J , Hoang K , et al. The calcium‐binding proteins S100A8 and S100A9 initiate the early inflammatory program in injured peripheral nerves. J Biol Chem. 2015;290:11771‐11784. 25792748 10.1074/jbc.M114.622316 PMC4416877

[41] Cesaro A , Anceriz N , Plante A , et al. An inflammation loop orchestrated by S100A9 and calprotectin is critical for development of arthritis. PLoS One. 2012;7(9):e45478. 10.1371/journal.pone.0045478 23029038 PMC3445527

[42] Ozes ON , Mayo LD , Gustin JA , et al. NF‐kappaB activation by tumour necrosis factor requires the Akt serine‐threonine kinase. Nature. 1999;401:82‐85. 10485710 10.1038/43466

[43] Damle SS , Moore EE , Nydam TL , et al. Postshock mesenteric lymph induces endothelial NF‐kB activation. J Surg Res. 2007;143:136‐140. 17950083 10.1016/j.jss.2007.04.016 PMC2128768

[44] Haasbach E , Reiling SJ , Ehrhardt C , et al. The NF‐kappaB inhibitor SC75741 protects mice against highly pathogenic avian influenza A virus. Antiviral Res. 2013;99:336‐344. 23811282 10.1016/j.antiviral.2013.06.008

