# Supplementary figures and images for: S100A9 induces nucleus pulposus cell degeneration through activation of the NF‐κB signaling pathway
Source: J Cell Mol Med. 2021 Mar 18;25(10):4709–20. doi: 10.1111/jcmm.16424 (PMC8107097; doi:10.1111/jcmm.16424)

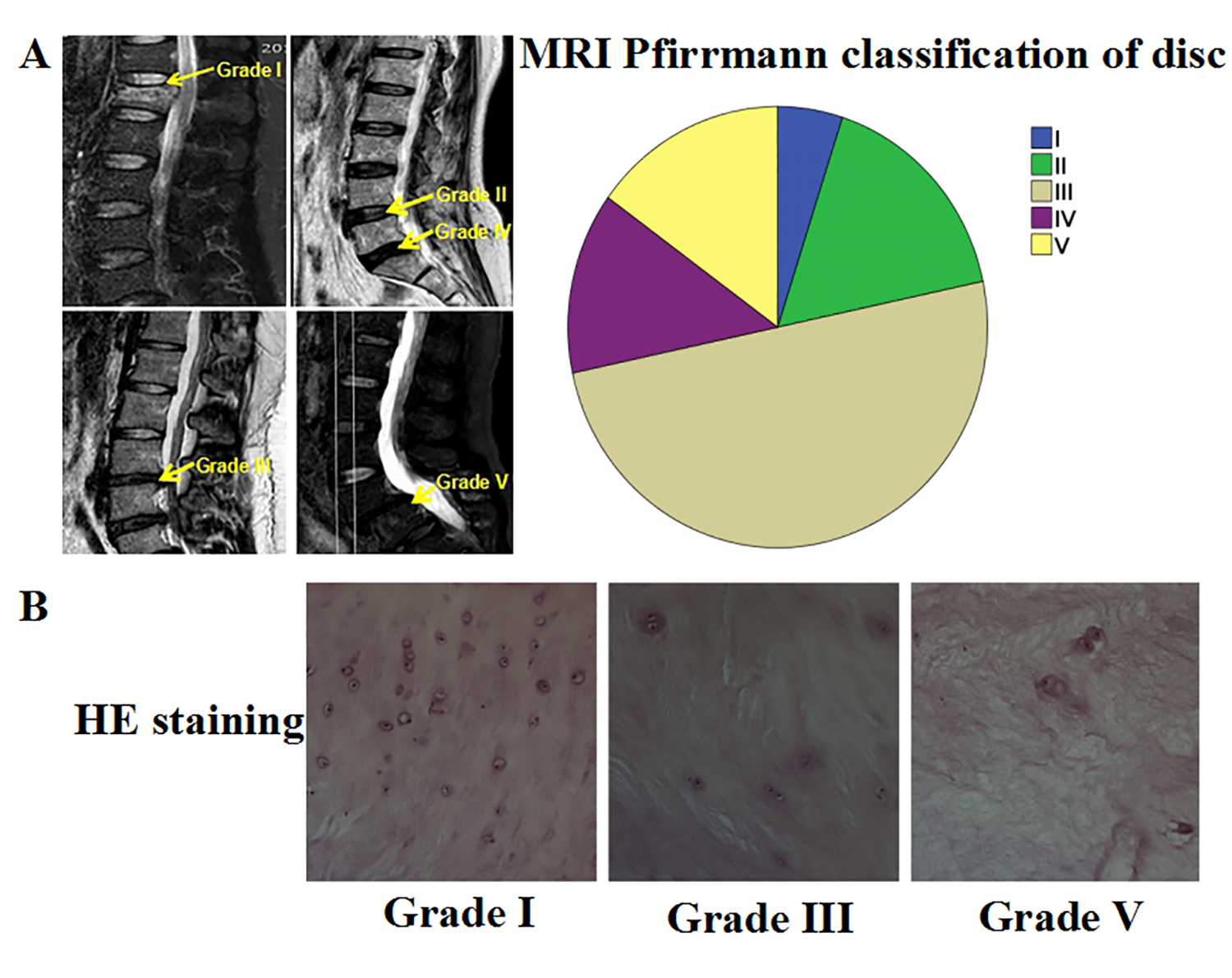

Supplement: Supplementary file 1 — Fig S1 [file JCMM-25-4709-s003.tif]

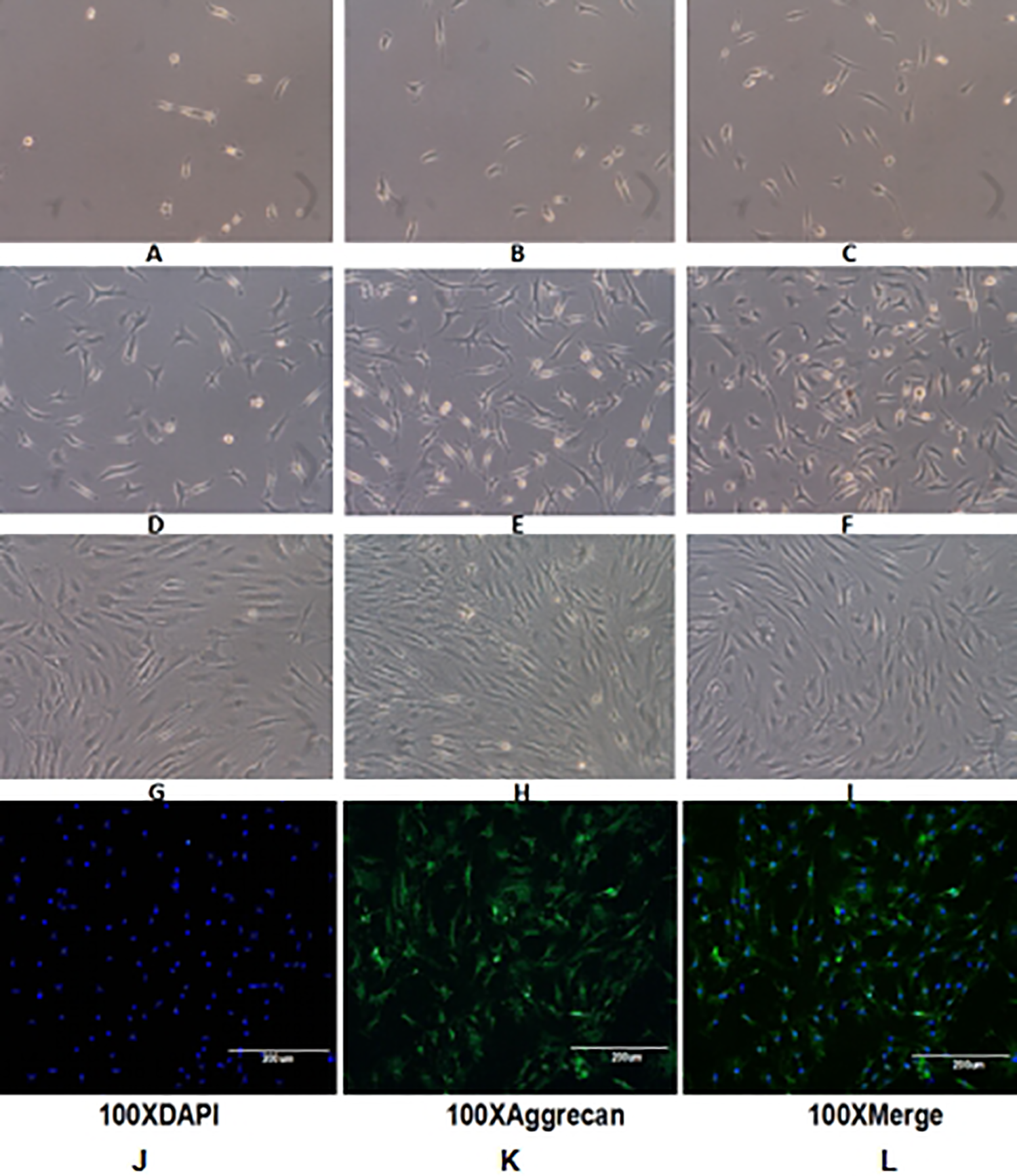

Supplement: Supplementary file 2 — Fig S2 [file JCMM-25-4709-s004.tif]

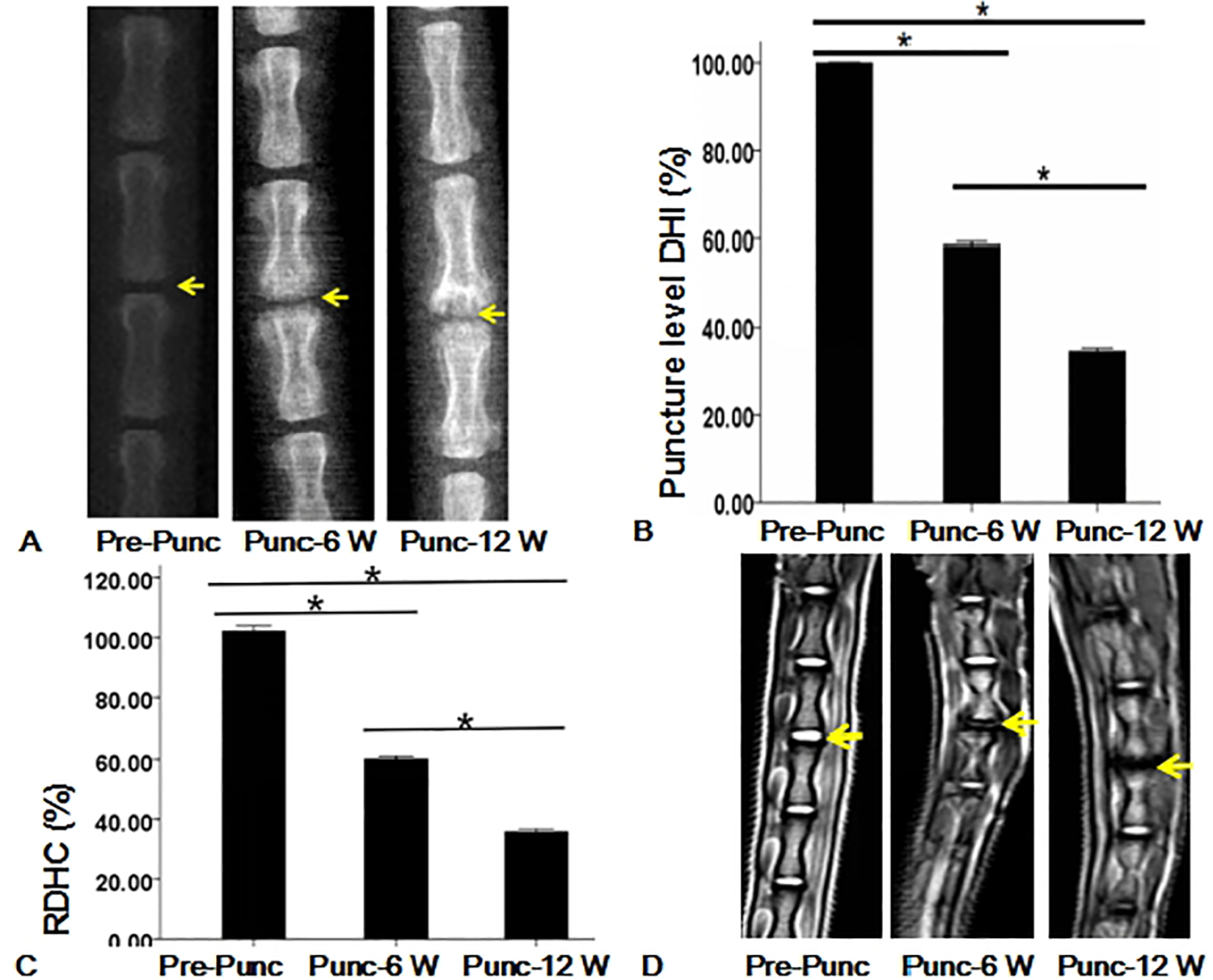

Supplement: Supplementary file 3 — Fig S3 [file JCMM-25-4709-s002.tif]

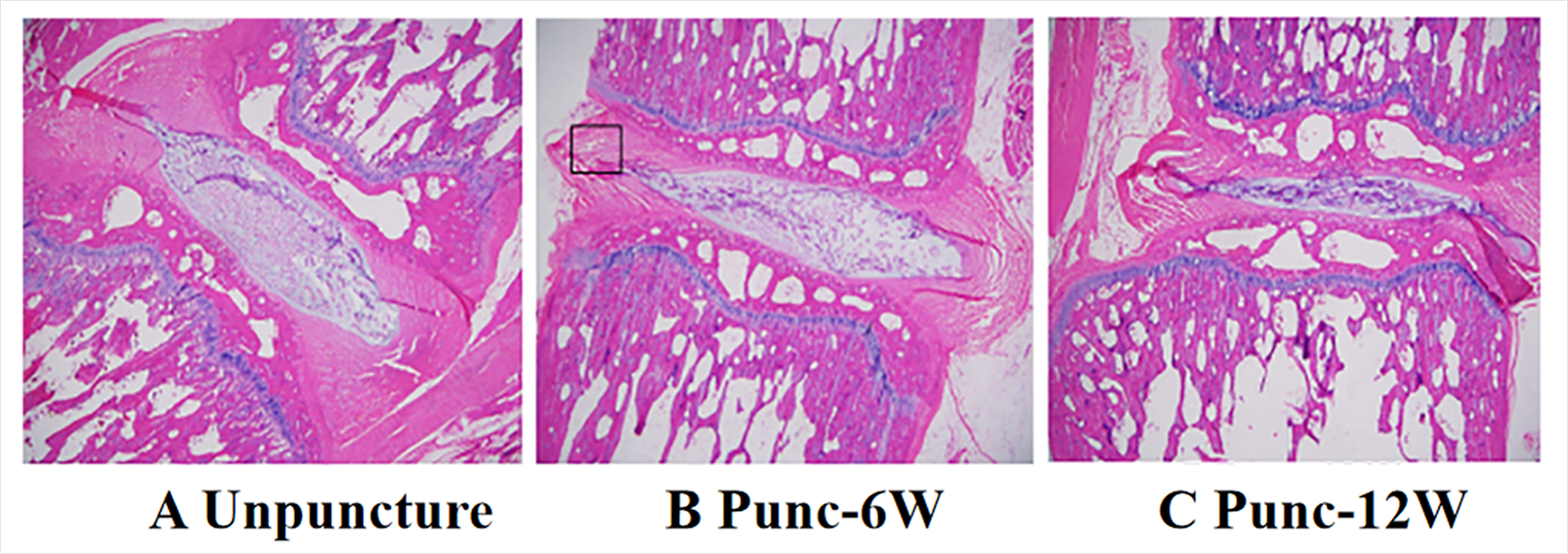

Supplement: Supplementary file 4 — Fig S4 [file JCMM-25-4709-s001.tif]
